# High-risk infant follow-up: where are we and where to from here?

**DOI:** 10.1038/s41390-025-04334-0

**Published:** 2025-08-29

**Authors:** Stephanie M. Boyd, Darazel Perez, Michelle Juarez, Nadia Badawi

**Affiliations:** 1 Grace Centre for Newborn Intensive Care, The Children’s Hospital at Westmead, Sydney, NSW Australia; 2 Specialty of Child & Adolescent Health, Sydney Medical School, Faculty of Medicine & Health, The University of Sydney, Sydney, NSW Australia; 3 Cerebral Palsy Alliance Research Institute, Specialty of Child & Adolescent Health, Sydney Medical School, Faculty of Medicine & Health, The University of Sydney, Sydney, NSW Australia

Diagnosing neurological complications in adults is significantly easier in comparison to newborn infants. Adults are able to describe their symptoms, such as headaches or weakness, thereby providing valuable clues as to their diagnosis, unlike newborns, who, in the absence of verbal communication, are reliant on the observation skills of clinicians. Adults often have clear signs, such as muscle weakness or loss of coordination, whereas signs in newborns may be non-specific and subtle, such as irritability or poor feeding. This is even more true for infants born preterm. Furthermore, adults have a baseline of normal neurological function, which serves to help detect deviations from the norm, unlike newborns whose nervous systems are still developing. Developmental immaturity can present difficulties in distinguishing between pathological conditions and normal developmental variation, particularly in premature infants. Imaging is also less straightforward in infants, who often require sedation, and in whom brain architecture is in evolution, with gestational age-specific features that complicate interpretation.

Consequently, neonates often receive a late diagnosis of neurological injury, and thus identifying who is at high risk is a challenging task on which to reach a consensus. The current paper by Clifford et al.^1^ highlights this dilemma, with respondents unable to agree on which high-risk infants require follow-up. Unsurprisingly, there was not unanimous agreement on the follow-up of preterm infants, although they represent nearly 50% of people who are subsequently diagnosed with cerebral palsy in high-income countries.^2^ As the authors indicate, it can be challenging to prognosticate in prematurity and multiple factors may interact to influence long-term outcomes for these infants. There was higher agreement on the need for follow-up for term infants who have had a stroke or neonatal encephalopathy, perhaps because they have more mature neurological systems than preterm infants, thereby allowing more consistent neurological responses. Or alternatively because the appearances of brain injury in these populations are so striking on neuroimaging, clinicians may subconsciously or consciously feel more compelled to ensure follow-up occurs. Although preterm infants may display obvious features of brain injury on imaging investigations that are predictive of outcomes, there exists a lack of consensus on the indications for magnetic resonance imaging in this patient group. National guidelines have been developed to stratify brain injury significance on cranial ultrasound, however, recommendations differ by region and definition and classification of the types of preterm brain injury vary.^3^ Additionally, in the absence of major neuroimaging abnormalities, serious red flags such as a requirement for neonatal surgery, multiple complications of prematurity, and microcephaly may not necessarily prompt the same sense of exigency, despite clinician awareness of their neurodevelopmental significance.

Another extremely vulnerable group are those infants with congenital heart disease and other surgical conditions. These infants are not routinely enroled in follow-up despite studies demonstrating they are at high risk of neurodevelopmental issues such as gross motor delays, as well as language, behaviour and cognitive problems.^4–6^ High rates of motor impairment and a significant risk of developmental coordination disorder at eight and nine years of age have also been described,^6^ however only 10% of respondents reported follow-up to eight years of age, with duration of follow-up most frequently reported until 24 months’ corrected age. This patient group often have genetic and chromosomal conditions and abnormal brain growth in utero. Furthermore, they undergo major procedures early in life involving several stimuli known to be injurious to the developing brain, including general anaesthetics, cardiopulmonary bypass, infections and low cardiac output states. Care of these infants is often shared between neonatal and paediatric intensive care units and the myriad opportunities for neurodevelopmental outcomes research in these groups may fall outside the typical scope of neonatology meetings with a perinatal focus. Both groups of infants have high rates of prolonged hospitalisation and intensive care unit admission, and their lack of explicit inclusion in follow-up recommendations, despite their established risk profile, is highlighted by Clifford and colleagues.

Socioeconomic factors are known to be a risk factor for developmental delay, including lower educational attainment. Recent studies have also shown that conditions such as cerebral palsy are also linked with socioeconomic deprivation. Mothers who live in deprived socioeconomic areas or have lesser educational qualifications are more likely to have a child with cerebral palsy (CP), and when they do, the child is more likely to require mobility equipment and have intellectual disability. Additionally, children who live in remote or regional Australia are also at higher risk of CP, as are children from First Nations families, 20% of whom live in remote or regional Australia compared with 2% of other Australians. Geographical remoteness can further negatively impact access to high-risk follow-up services. However, in the current study, around half of respondents did not consider socioeconomic deprivation to be an indication for follow-up. While respondents could identify individual socioeconomic risk factors such as being a teenage mother or using drugs of dependency, it was unclear whether they factored these findings into follow-up planning. Physical health was seen to be consistently prioritised over socioeconomic factors. The effect of this is likely to be exacerbated in low- and middle-income countries, many with large populations, who face higher rates of neonatal morbidity, and both a greater burden of social disadvantage and less robust systems and resources for follow-up.

In addition to identifying and addressing additive socioeconomic risk, there exists in general a lack of emphasis on opportunities for neurodevelopmental support, other than formalised assessments, for NICU graduates and their families. Support for parental mental health, education around neurodevelopmentally supportive caregiving, and environmental enrichment are examples of interventions that empower parents to optimally support their vulnerable children at home. This concept of ‘follow-through’ longitudinal parental support beyond medical and technical aspects is addressed by the current paper and should ideally be integrated with follow-up services. The authors describe the potential value of machine learning in developing logarithms to optimise follow-up programs, and this is already being studied in General Movements assessment analysis to increase its efficiency and applicability to population screening.

Interestingly, the specialists did not agree on the factors that increased the risk of neurodevelopmental disability, with some emphasising clinical picture or course and others emphasising neuroimaging. The potential for individualised prognostication for specific outcomes with the use of a multimodal approach incorporating a combination of these, as well as aEEG was highlighted. In practice, this difficulty in deciding who requires follow-up persists and translates into a loss of opportunity to maximise children’s full potential. Cerebral palsy is an exemplar condition and data from the Australian Cerebral Register indicate that only 21% of children are diagnosed in the first 6 months of life,^1^ with the remainder missing out on intervention during the crucial time of maximal brain growth and possible neurorehabilitation. In contrast, most adults who have a stroke have clinically obvious signs, and the current recommendations are to institute therapy in the first three days post-injury and to provide active early intervention, which results in improved speech, fine motor and gross motor outcomes.^7^

So why should this matter? Parents of infants in the NICU or special care nursery know that their infants are at high risk of neurodevelopmental and behavioural problems. They tell us that early diagnosis helps reduce the stress and uncertainty they feel. It increases clarity regarding their child’s condition and helps them receive early support.^8^ The definition of this early diagnosis and early support continues to be modified in light of evolving evidence for best practice. In many NICUs, General Movements assessments are routinely performed both during hospital admission and as an important component of follow-up post discharge. We have altered our follow-up timing to review fidgety movements at around 13 weeks post-term age to facilitate referral to Early Diagnosis Clinics (EDCs), which accelerate diagnosis for infants at high risk of CP. The EDCs are recognised as global best-practice for CP diagnosis and feature a specialist multidisciplinary team of clinicians, inclusive of a paediatric neurologist and social worker. Additionally, randomized controlled trial data show that infants with unilateral/hemiplegic cerebral palsy who receive early Constraint Induced Movement Therapy (CIMT) at 3 to 5 months of age have better hand function than controls in the short- and long-term. Ongoing structural changes in the corticospinal system are directed to hand function, with these observed changes shown to be activity-dependent. For every dollar spent on early intervention, you get at least a threefold return, and early intervention in preterm infants improves cognitive outcomes until school age.

With early detection of cerebral palsy now the standard of care in Australia and some other high- income countries, two randomised pilot studies of early intervention between 2013 and 2015 have demonstrated promising results in both motor and cognitive outcomes, adjusting for severity of brain injury and visual impairment in infants with CP. RCT data are also beginning to indicate that infants with any type and topography of cerebral palsy, who receive “GAME” (Goals – Activity – Motor Enrichment, which is an early, intensive, enriched task-specific, training-based intervention at home), have better motor and cognitive skills at one-year than those who received usual care.^9^ The guidelines for early detection published in 2017^10^ advocate bringing forward diagnosis and improving intervention options for infants with brain injuries during the period of rapid brain growth, with the ultimate aim of harnessing experience-dependent plasticity. In addition to neurorehabilitation, we know from CPUP Register data from Sweden that children with bilateral cerebral palsy who receive regular surveillance and intervention have lower rates of: hip displacement, contractures and scoliosis. Thus, early diagnosis carries with it preventative health benefits beyond optimising brain function.

High-risk infant follow-up programs are crucial for monitoring and supporting infants who have experienced neonatal complications. However, several challenges exist in current practice. The paper by Clifford and colleagues^1^ highlights the problems with the variable criteria for follow-up and how these lead to inconsistencies in care. These inconsistencies are largely driven by the fact that there are limited resources for follow-up and a lack of skilled therapists, which limits access to a Multidisciplinary Care model. Ideally, follow-up should involve specialists such as neonatologists, psychologists, physiotherapists, occupational therapists, speech therapists and nutritionists, however access to such comprehensive teams is not always available. This limited access can have several serious implications, including late diagnosis of developmental delays, or neurological issues going unnoticed, leading to missed opportunities for early intervention therapy for infants and support services for families. Inconsistent care practices, barriers to access and lack of coordination between different healthcare providers can result in fragmented care, where infants do not receive timely comprehensive intervention, resulting in high long-term costs as issues are not addressed until they become severe. Without access to multidisciplinary teams, infants may require more intensive medical and educational support later in life. Parents struggle to be heard and to navigate complex healthcare systems, which can lead to significant financial and emotional strain, and effects on the well-being of the wider family unit.

Improving access to multidisciplinary care in high-risk infant follow-up requires a combination of systemic changes and targeted interventions, including standardized follow-up protocols that allow for consistent assessment schedules and criteria throughout the healthcare systems that ensure all infants receive appropriate monitoring. Investing in enhanced telemedicine services and virtual consultations can help bridge gaps in access, especially for families in remote areas.

In conclusion, it is imperative that health care professionals and developmental specialists must lobby governments to understand the value of early detection and early intervention programs. A universal approach may be challenging. The framework of high-risk infant follow-up must be tailored to reflect the workforce capacity, local resource availability and population needs. This will require increased funding and policy changes to allocate resources to expand access to multidisciplinary teams and improve infrastructure. It will be money well spent.
